# The Effects of ADHD Teacher Training Programs on Teachers and Pupils: A Systematic Review and Meta-Analysis

**DOI:** 10.1177/1087054720972801

**Published:** 2020-12-17

**Authors:** Rebecca J. Ward, Sarah J. Bristow, Hanna Kovshoff, Samuele Cortese, Jana Kreppner

**Affiliations:** 1 University of Southampton, Southampton, Hampshire, UK; 2 Solent NHS Trust, Southampton, UK; 3 New York University Child Study Center, New York, NY, USA; 4 University of Nottingham, Nottingham, UK

**Keywords:** ADHD, teacher, pupil, attention-deficit/hyperactivity disorder, knowledge, behavior

## Abstract

**Objective:**

To synthesize the evidence on the efficacy of ADHD teacher training interventions for teachers’ ADHD knowledge and reducing pupils’ ADHD-type behaviors.

**Method:**

Six electronic databases were systematically searched up to 14/04/20. Meta-analyses were performed to pool standardized mean differences (SMD).

**Results:**

29 studies were included in the systematic review, and 22 meta-analyzed. SMD for teacher knowledge within subjects at post-test and follow-up was 1.96 (95% confidence interval = 1.48, 2.43) and ‒1.21 (–2.02, –0.41) respectively. Between subjects analyses at post-test showed SMD = 1.56 (0.52, 2.59), with insufficient data at follow-up. At post-test, SMD for pupils’ behavior within and between subjects was 0.78 (0.37, 1.18), and 0.71 (–0.11, 1.52), respectively. Medium-to-high risk of bias was found in all but one study.

**Conclusion:**

ADHD teacher training programs may be effective in initially improving ADHD teachers’ knowledge. There is inconsistent evidence for their efficacy to reduce students’ ADHD-type behaviors.

ADHD is one of the most commonly diagnosed childhood conditions. Meta-analytically pooled data (Polanczyk et al., 2007; Thomas et al., 2015) provide estimates of 5%–7% (95% CI = 5.01–5.56; 6.7–7.8 respectively) in school-aged children, equating to approximately one child per classroom (Dalsgaard et al., 2014) and if left untreated, can lead to significant, functional impairments. The prevalence rate in adults is estimated to be 2.5% (95% CI = 2.1–3.1; Simon et al., 2009) evelopmentally inappropriate levels of inattention and/or impulsivity-hyperactivity create problems in school, disrupting learning and peer relationships (American Psychiatric Association, 2013; Daley & Birchwood, 2010; Loe & Feldman, 2007). The classroom behavior of children with ADHD can also negatively impact learning for other students and teachers (DuPaul & Stoner, 2016; Wheeler & Carlson, 1994). Academic underachievement for children with ADHD can have lifelong implications associated with poor academic and vocational progression, social skills and relationships, poor mental health, and criminality (Langberg & Becker, 2012; Montgomery et al., 2018; Parker et al., 2013), yet few studies investigating teacher training interventions report follow-up measures to show long-term effects; those that do are limited to 6 months post-intervention (e.g., Both et al., 2016) making it difficult to assess the long-term benefit of the training. Given that the average child spends over 13,000 hr in compulsory school education (Long, 2019; Rutter, 1979), it is critical to find effective interventions in schools to support children with ADHD.

One of the main treatment recommendations for ADHD, alongside pharmacological treatment, involve behavioral interventions (National Institute for Health and Care Excellence, 2019; Pfiffner & Dupaul, 2015; Wolraich et al., 2011, 2019). Researchers have demonstrated that teachers’ knowledge of ADHD significantly correlates with teachers’ confidence in their ability to effectively teach children with ADHD, create an inclusive classroom and manage behavior (Bussing et al., 2002; Ohan et al., 2008; Sciutto et al., 2000). Furthermore, diagnostic processes rely greatly on teachers’ information on children (Topkin et al., 2015; Wolraich et al., 2003); in fact, teachers are often the first to identify behavioral difficulties (Both et al., 2016; Shelemy et al., 2019). Therefore, with early referral being key to address problem behaviors before they become well-established (Aguiar et al., 2014) it is vital for teachers to have appropriate knowledge of ADHD so they can recognize and act on symptoms early.

ADHD teacher training interventions have been developed to strengthen teachers’ knowledge about ADHD, train them to create a supportive environment in the classroom, and develop strategies to address problem behaviors. Studies investigating teachers’ knowledge of ADHD and its impact on teaching behaviors, identify a need for more continuing professional development to address knowledge gaps (Bekle, 2004; ComRes, 2017; Sciutto et al., 2016), better quality training for education students (Bekle, 2004; Kos et al., 2004), and further research into classroom management techniques and curriculum planning (Bekle, 2004; Kołakowski et al., 2009; Shelemy et al., 2019). A systematic review of studies measuring teachers’ ADHD knowledge conducted by Mohr-Jensen et al. (2019), found knowledge scores varied considerably for symptoms, behaviors, prognosis and treatment, and identified educating teachers about ADHD as a key factor in raising knowledge levels. The majority of specific teacher training programmes for ADHD have focused on increasing knowledge and shown these programmes to be effective (Aguiar et al., 2014; Anto & Jacob, 2014; Syed & Hussein, 2010).

While many teacher training programmes also include behavioral management strategies, few studies report improvements in teachers’ use of positive behaviors toward children with ADHD, and with the exception of Park and Park (2017), date from over ten years ago (Bloomquist et al., 1991; Miranda et al., 2002; Rossbach & Probst, 2005). In this context, it is important to recognize that teachers are typically reluctant to endorse more intensive management strategies which impinge on planning and preparation or require additional staff within the classroom. Instead they tend to use less intensive strategies more frequently, for example: breaking verbal instructions down into simple, step-by-step patterns; positive teacher feedback; and creating seating plans in the classroom (Blotnicky-Gallant et al., 2014). However, Kos (2008) suggests that a lack of consistency in implementing good strategies repeatedly with the same child can result in little behavior change for that child.

Effects of teacher and classroom strategies on the ADHD-type behaviors of pupils in the classrooms are also measured in relatively few studies (e.g., Bloomquist et al., 1991; Corkum et al., 2019; Froelich et al., 2012). This is, perhaps, surprising given the literature suggests that the rationale for teacher training in ADHD, in addition to improving self-efficacy and self-confidence for teachers, is to improve the social and educational outcomes of the child with ADHD (Anto & Jacob, 2014; Barnett et al., 2012).

A systematic understanding of the effectiveness of reported ADHD teacher training programmes is compromised by the fact that comparison across studies is difficult because a variety of outcome measures and methodologies are used (Norris & Atkins, 2005; Reed et al., 2005) which span different professional sectors, namely, psychological, medical, and educational (Singh, 2011; Smith, 2017). Firstly, there are few randomized controlled trials (RCTs) and significant heterogeneity in study designs (Deeks et al., 2003; Norris & Atkins, 2005). The majority of studies investigating ADHD teacher training interventions are non-randomized studies, including many single-arm cohorts (Latouche & Gascoigne, 2019; Lessing & Wulfsohn, 2015; Shehata et al., 2016). In addition, these studies vary in terms of design, intervention characteristics, heterogeneous recruitment techniques, measurement tools, and measurement timeframes (Anto & Jacob, 2014; Corkum et al., 2019; Lasisi et al., 2017). Secondly, there are only few well-developed tools to assess risk of bias in non-randomized studies (Deeks et al., 2003; Reed et al., 2005), particularly when a number of different study designs are included (Deeks et al., 2003; Stang, 2010; Sterne et al., 2016). Thirdly, outcome measures of symptom change in children following teacher training tend to be completed by participating teachers, raising the risk for bias in measurement of outcomes (Sterne et al., 2016). Finally, fidelity to the intervention is important when assessing its effectiveness in order to accurately assess the impact of the intervention as it was designed and to be able to replicate findings in other groups and yet rarely reported (Johnson et al., 2006; McKenna et al., 2014).

With the methodological limitations of the literature in mind, the present study aims to provide a rigorous systematic review and meta-analysis of the available evidence for the effectiveness of ADHD teacher training interventions. To our knowledge, there has been no published quantitative synthesis of the literature specifically focused on the efficacy of ADHD training for qualified teachers to improve knowledge on ADHD as well as reduce pupils’ ADHD-type behaviors of hyperactivity, impulsivity and inattention.

The following questions guided the present systematic review and meta-analysis:

Primary question: How effective are ADHD teacher training interventions in increasing teachers’ knowledge and positive behaviors toward children with ADHD-type behaviors?
Secondary question: Does an ADHD teacher training intervention result in reduced ADHD-type behaviors of pupils in the classrooms of participating teachers?

Given the exploratory nature of the meta-analysis, no a priori hypotheses were formulated.

## Method

This systematic review and meta-analysis was conducted according to the PRISMA recommendations (Preferred Reporting Items for Systematic Reviews and Meta-Analyses; Moher et al., 2009). The protocol for this review and meta-analysis was pre-registered in PROSPERO (# removed to preserve anonymity).

### Search Strategy

Initially, on November 8, 2019, a systematic search was performed in six electronic databases (covering medical, educational and psychology domains): PsycINFO, CINAHL Plus, ERIC, MEDLINE (EBSCO), Web of Science, and Scopus. Search terms were defined using the PICO format (see Table 1). Additionally, backward and forward citation chasing were conducted. Peer-reviewed studies and gray literature were included to avoid selection or publication bias. Similarly, no language or date restrictions were placed on the search to avoid these biases. A final search was conducted on April 14, 2020 to capture any articles published between the initial search and submission for publication. This search revealed no new studies that met the inclusion criteria.

**Table 1.** PICO Search Terms.

Participant	(Teacher* OR Educator* OR “Educational practitioner*” OR Schoolteacher* OR Pupil* OR Student* OR Learner* OR Teen* OR Child* OR “Young people” OR Adolescen* OR Youth* OR Infant* OR Junior*)
Intervention	(“Training program*” OR “school-based” OR CPD OR “Professional development” OR Psychoeducation OR “In-service training” OR “Incredible Years” OR Triple-P OR “Coaching program*” OR “teacher training” OR “teacher program*” OR “in-service teacher education” OR “teacher education”)
Condition	(ADHD OR AD/HD OR “Attention-deficit/ hyperactivity disorder” OR “Attention deficit hyperactivity disorder” OR “Attention deficit disorder” OR “hyperkinetic disorder” OR Inattent* OR Hyperactiv* OR overactiv* OR off-task OR “Emotional Behavioral Disorder” OR “Emotional Behavioral Difficulty”)
Outcome	(Attitude* OR Behavio* OR Skill* OR “Classroom management” OR Knowledge OR Effectiveness OR Efficac* OR Impact OR Symptom* OR Strateg* OR Attainment OR Progress OR Achievement)

### Inclusion and Exclusion Criteria

Inclusion and exclusion criteria were determined to address the research questions (see Table 2). Teacher training interventions that were primarily or solely comprised of psychoeducation and/ or behavioral strategies to address ADHD specifically were the focus of this review and meta-analysis, and interventions where ADHD formed a minor part of the content, or more broadly focused interventions for problem behaviors, were excluded. If the study sample included a mixture of teachers from both mainstream and special education settings, the study was only included if it was possible to obtain and extract the data for mainstream teachers only.

**Table 2.** Inclusion and Exclusion Criteria.

Criteria	Inclusion	Exclusion
Population	Primary or Secondary School teachers	Pre-school teachers, post-compulsory education teachers, teaching assistants, other educational professionals, teachers in special schools
	Children with a diagnosis of ADHD or identified as displaying ADHD-type behaviors (i.e., hyperactivity, impulsivity, inattention/ off-task behavior)	
	Children in primary or secondary mainstream education (aged 4–16 years)	Children in special schools, children in pre-school or post-16 education
Intervention	ADHD teacher training interventions for in-service teachers (of any type, delivery mode, duration or intensity)	Teacher training interventions delivered prior to teacher qualification for example, in teacher training colleges.
	ADHD teacher training interventions which have one condition as teacher training only	Training interventions where the teacher component is combined with other groups for example, parents, child
		Training interventions where ADHD is a minor component of the training, for example, induction training, or one part of a larger training programme.
Comparison	No comparison group, waitlist control, alternative treatment, control group	
Outcome	For teachers in mainstream primary and secondary classrooms:	Measures for special education teachers
	• measures of teachers’ ADHD knowledge	
	• measures of teachers’ behavior management strategies toward children with ADHD and ADHD-type behaviors	
	For children with a diagnosis of ADHD or identified as displaying ADHD-type behaviors (i.e., hyperactivity, impulsivity, inattention/ off-task behavior) in primary or secondary education:	Measures for children in special schools, pre-school or post-16 education
	• measures of child ADHD symptoms (e.g., inattention including off-task behaviors, impulsivity, hyperactivity) and related impairments, including problem behaviors and social functioning	
Study design	Controlled trials (randomized and non-randomized), intervention studies	Qualitative studies
Date	All dates included	
Location	Global	No locations excluded
Language	All languages (if translation is possible)	No languages excluded unless translation not possible due to time or financial constraints
Types of publication	Peer-reviewed journal articles and gray literature (dissertation theses, reports, articles in press)	Any other type of publication, including conference papers
Databases	Six electronic databases were searched encompassing psychology, education and medical literature: PsycINFO, CINAHL Plus, ERIC, MEDLINE (EBSCO), Web of Science, Scopus	Any other databases
Terms (plus synonyms detailed in the PICO document)	Teacher	
	Pupil	
	ADHD	
	Training	
	Teacher knowledge, teacher behavior	
	Child ADHD symptoms	

### Screening and Study Selection

**Figure 1.**
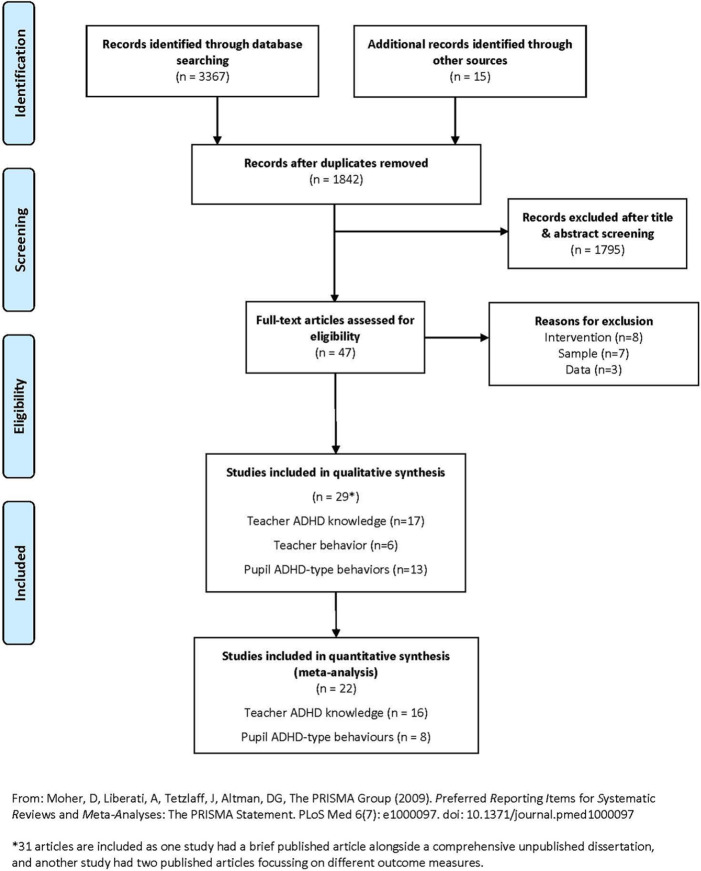
PRISMA diagram.

The results of the database searches were exported to Endnote X9 and duplicates were removed. Titles and abstracts of the remaining studies were then screened and non-pertinent papers removed. Full-text screening was conducted on the remainder to identify the studies to be included in the systematic review. These were further screened for inclusion in the meta-analysis determined by whether sufficient data were reported to calculate effect sizes at pre-test and post-test points, and follow-up, if appropriate (see Figure 1). Where there was insufficient data available in published articles, study authors were contacted up to two times.

Each stage of the literature search and screening process was undertaken by two independent researchers (initials removed for anonymity) and any conflicts were resolved through discussion and consensus. A third independent, senior researcher (initials removed for anonymity) was available to make a final decision in the event of no resolution.

### Data Extraction

Selected studies were initially organized by outcome measures. Two groups were formed: teacher outcomes and pupil outcomes. Teacher outcomes were divided into two subgroups: teacher knowledge and teacher behavior strategies. Pupil outcomes measured pupil behavior related to ADHD symptoms. The following data were manually extracted from each study by two independent researchers and recorded in Microsoft Excel: intervention content (topics) and mode of delivery (e.g., face-to-face, online) and length of intervention (e.g., number of sessions, duration of sessions), numbers of participants (intervention group and any comparison group), and the outcome measures reported for each group in the study (see Supplemental Appendix 1).

### Outcome Measures

The following outcomes were included in the analysis: (a) teacher ADHD knowledge, measured with self-report questionnaires (b) teacher behaviors toward pupils with ADHD-type behaviors, measured with a variety of tools including self-report using vignettes, self-report questionnaire and blinded observations (c) pupil ADHD-type behaviors tested with a variety of measures including observations and teacher reports. For studies that reported pupil ADHD-type behaviors with more than one measure, a hierarchy was established before extracting the data. This hierarchy ensured the most proximal assessment, which was a report by the rater closest to the classroom setting (i.e., the teacher) of hyperactivity, impulsivity and inattention. If more than one measure was used by the teacher, the hierarchy was based on the validity and reliability of the tools used (see Supplemental Appendix 2).

### Risk of Bias

Risk of bias for the selected studies was assessed independently by two researchers using the revised Cochrane Risk of Bias Tool (ROB2; Higgins et al., 2019) for randomized controlled trials, and the Risk of Bias for Non-randomized Studies of Interventions (ROBINS-I; Sterne et al., 2016) for all other studies. Global risk of bias for each study was calculated by the instructions supplied for each tool; namely, that an overall medium or high risk of bias was determined if a medium or high risk of bias was found in any one domain, respectively.

A list of confounding variables was compiled by the research team (Table 3) to complete the risk of bias for non-randomized studies. Disagreements were resolved by discussion and agreement within the research team.

**Table 3.** Confounding Variables for Non-Randomized Studies.

Teacher outcome measures	Pupil outcome measures
**Characteristics of teachers**	**Characteristics of pupils**
Previous knowledge of ADHD	Age of child
Years of teaching	Gender of child
ADHD medication for children	Severity of ADHD
Contamination if teachers from the same school are in both the intervention and control groups	Comorbidities
Experience of management of student with ADHD	ADHD medication for children
**Characteristics of delivery**	**Characteristics of delivery**
Duration and mode of delivery within study	Duration and mode of delivery within study
**Characteristics of the school/ setting**	**Characteristics of the school/ setting**
Differences between schools	Differences between schools

### Analytic Plan

The meta-analysis was conducted using Comprehensive Meta-Analysis, which allowed for effect size data to be entered in multiple formats, including means and standard deviations, paired *t*-tests and correlations (Borenstein et al., 2014). Due to the different types of behaviors measured (e.g., punitive reactive strategies, labeled praise, rule violations by pupils) and the range of tools used (including blinded observations, self-report of intended teacher behavior using vignettes, self-report of actual teacher behavior), effects for change in teacher behavior strategies were not meta-analyzed. Analyses were conducted for pre-test to post-test measures to investigate the effects of the intervention, and from post-test to follow-up to examine whether any improvements at post-test were sustained at follow-up. For post-intervention outcomes, standardized mean differences (SMD) for effect measures with a 95% confidence interval were calculated, and a random-effects model was used due to the expected heterogeneity between studies. A chi-squared test and the I-squared statistic assessed heterogeneity, with an I-squared value greater than 50% suggestive of substantial true (as opposed to random) heterogeneity. Publication bias was measured, using funnel plots and Egger’s test, for any analysis comprising ten or more studies (Higgins et al., 2019). Subgroup meta-analyses to compare the results from randomized controlled trials to non-randomized studies, as well as interventions for primary teachers and secondary teachers, were planned in order to investigate possible moderators of effects.

## Results

The systematic search identified 29 studies conducted in 18 countries: Australia (*n* = 1), Brazil (*n* = 1), Canada (*n* = 3), Egypt (*n* = 1), Ethiopia (*n* = 1), Germany (*n* = 4), India (*n* = 1), Iran (*n* = 2), Netherlands (*n* = 1), Nigeria (*n* = 1), Pakistan (*n* = 1), Poland (*n* = 1), Saudi Arabia (*n* = 1), South Africa (*n* = 1), South Korea (*n* = 1), Spain (*n* = 1), Turkey (*n* = 1) and the United States (*n* = 6). Twenty-two studies provided sufficient data for meta-analysis. Seven studies required translation into English from the following languages: Arabic, French, German, Korean, Polish, and Turkish.

### Study Design and Participant Information

Of the 29 retained studies, ten were randomized controlled trials and 19 non-randomized studies (see Table 4), including non-randomized controlled trials (*n* = 5), uncontrolled before-and-after comparison studies (*n* = 13), and one multiple-baseline trial. Sample sizes ranged from 6–150 participants, comprising a mix of primary (*n* = 26) and secondary teachers (*n* = 3), and children with a clinical diagnosis of ADHD (*n* = 4) as well as those displaying ADHD-type behaviors at sub-clinical levels (*n* = 7).

**Table 4.** Overview of Included Studies (RCTs in Bold) Including Interventions and Measures.^a^.

Study no.	First Author (year)	Study design	Sample *N*	Comparison group(s)	Content of training	(Mode) & duration of training	Primary Outcome	Most Proximal Assessment
1	Aguiar (2014)	Uncontrolled before-and-after design	37 teachers	None	Psychoeducation, Behavioral Strategies	(Face-face) 1 × 6 hour session	Teacher knowledge	Study own questionnaire, teacher
2	Anto (2014)	Uncontrolled before-and-after design	50 teachers	None	Psychoeducation, Behavioral Strategies	(Self-instruction booklet) 1 week	Teacher knowledge	Study own questionnaire, teacher
3	Barbaresi (1998)	Uncontrolled before-and-after design	44 teachers	None	Psychoeducation, Behavioral Strategies	(Face-face) 1 × 2.5 hour session	Teacher knowledge	Study own questionnaire, teacher
4	Barnett (2010) ^bc^	Uncontrolled before-and-after design	19 teachers	None	Psychoeducation, Behavioral Strategies	(Self-instruction online) 7 weeks	Teacher knowledge	KADDS, TBQ, teacher
							Teacher behavior	
5	Barnett (2012)^c^	Uncontrolled before-and-after design	19 teachers	None	Psychoeducation, Behavioral Strategies	(Self-instruction online) 7 weeks	Teacher knowledge	KADDS, TBQ, teacher
							Teacher behavior	
**6**	**Bloomquist (**1991**)**	RCT (multiple-armed)	12 ADHD children	13 ADHD children control, 11 multicomponent condition	Psychoeducation, Behavioral Strategies	(Face-face) 2 × 1 hour session	Pupil behavior	Blinded observation
						10 × 1 hour consultation		
7	Both (2016)	Uncontrolled before-and-after design	44 teachers	None	Psychoeducation, Behavioral Strategies	(Face-face) 1 × 2.5 hour session	Teacher knowledge	KADDS teacher
**8**	**Corkum (**2019**)**^f^	RCT	28 teacher, ADHD pupil^e^ dyads	30 waitlist control teacher/ student dyads	Psychoeducation, Behavioral Strategies	(Self-instruction online) 6 weeks	Pupil behavior	Conners 3-T teacher
9	Froelich (2012)	Non-randomized controlled trial	8 teachers, 25 ADHD children	8 teachers	Psychoeducation, Behavioral Strategies	(Face-face) 12 × 2 hour sessions	Pupil behavior	YCI teacher
				17 children				
10	Gormley (2015) ^f^	Multiple baseline design	3 teacher, ADHD children dyads	None	Behavioral Strategies	(Face-face) 2 years biweekly	Pupil behavior	BOSS, blinded
11	Kołakowski (2009)	Uncontrolled before-and-after design	150 teachers	None	Psychoeducation, Behavioral Strategies	(Face-face) 15 hour over 3 months	Teacher knowledge	Study own questionnaire, teacher
**12**	**Lasisi (**2017**)**	RCT	84 teachers	75 waitlist control teachers	Psychoeducation, Behavioral Strategies	(Face-face) 1 × 2.5 hour session	Teacher knowledge	SRAQ teacher
13	Latouche (2019)	Non-randomized controlled trial	113 teachers	161 waitlist control teachers	Psychoeducation, Behavioral Strategies	(Face-face) 1 × 2hour session	Teacher knowledge	KADDS teacher
14	Lauth-Lebens (2016)	Uncontrolled before-and-after design	25 teachers/25 ADHD children	None	Psychoeducation, Behavioral Strategies	(Face-face) 7 × 90 minute sessions	Pupil behavior	DSM-IV-TR symptom list teacher
15	Lessing (2015)	Uncontrolled before-and-after design	1 teacher/10 ADHD children^e^	None	Behavioral Strategies	(Face-face) Not reported	Pupil behavior	CTRS-R teacher
**16**	**Miranda (**2002**)**	RCT	29 teachers/29 ADHD children	21 teachers/21 ADHD children	Psychoeducation, Behavioral Strategies	(Face-face) 8 × 3 hour sessions+ 8 weekly interviews	Teacher knowledge	Study own questionnaire, teacher, Non-blinded observation, teacher
							Pupil behavior	
17	Mohammed (2018) ^f^	Non-randomized controlled trial	9 children with ADHD symptoms	9 normative children	Psychoeducation, Behavioral Strategies	(Face-face) 6 × 6 hour sessions + weekly coaching	Pupil behavior	BOSS, blinding unknown
18	Nadeau (2012)^f^	Non-randomized controlled trial	11 teachers	26 teachers	Psychoeducation, Behavioral Strategies	(Face-face) 6 × 2 hour coaching	Teacher behavior	Study own questionnaire, teacher
**19**	**Obaidat (**2014**)**	RCT	40 teachers	40 teachers	Psychoeducation, Behavioral Strategies	(Face-face) 8 × 2 hour sessions	Teacher knowledge	Study own questionnaire, teacher
**20**	**Owens (**2017**)**	RCT	31 teachers	27 teachers	Behavioral Strategies	(Face-face) 1 × 3 hour session, 8 × 30 minute coaching	Teacher behavior	Blinded observation
21	Park (2017)	Non-randomized controlled trial	35 teachers	35 teachers	Psychoeducation, Behavioral Strategies	(Face-face), 8 × 1 hour sessions	Teacher knowledge	KADDS, PSEIA, K-ARS, teacher
							Teacher behavior	
							Pupil behavior	
22	Procaccini (2014) ^b^	Uncontrolled before-and-after design	35 teachers	None	Psychoeducation	(Self-instruction online) 1 × 45 minute session	Teacher knowledge	KADDS, teacher
23	Rossbach (2005)	Uncontrolled before-and-after design	6 teachers, 6 ADHD children	Teachers *n* = 5, 5 ADHD children	Psychoeducation, Behavioral Strategies	(Face-face) 3 × 4 hour sessions	Teacher knowledge	Study own questionnaire, teacher, DSM-IV symptom list, teacher
							Pupil behavior	
**24**	**Sarraf (**2011**)**	RCT	35 teachers	35 teachers	Psychoeducation, Behavioral Strategies	(Face-face) 2 × day sessions	Teacher knowledge	Study own questionnaire, teacher
**25**	**Shaban (**2015**)**	RCT	32 ADHD children^e^	32 ADHD children	Behavioral Strategies	(Face-face) 8 x 3hour sessions	Pupil behavior	TRF, teacher
26	Shehata (2016)	Uncontrolled before-and-after design	60 teachers	None	Psychoeducation, Behavioral Strategies	(Face-face) 15 × 1 hour sessions	Teacher knowledge	KADDS, TBSS, teacher
							Teacher behavior	
27	Syed (2010)	Uncontrolled before-and-after design	49 teachers	None	Psychoeducation, Behavioral Strategies	(Face-face) 5 × 2 hour sessions	Teacher knowledge	Study own questionnaire, teacher
28	Tahiroğlu (2004)	Uncontrolled before-and-after design	104 teachers	None	Psychoeducation, Behavioral Strategies	(Face-face) 1 × 2 hour session	Teacher knowledge	Study own questionnaire, teacher
**29**	**Veenman (**2017**)**^d,f^	RCT	58 children	56 children	Psychoeducation, Behavioral Strategies	(Face-face) 18 week program	Pupil behavior	COC, non-blinded
**30**	**Veenman (**2019**)**^d,f^	RCT	58 children	56 children	Psychoeducation, Behavioral Strategies	(Face-face) 18 week program	Pupil behavior	COC, non-blinded
**31**	**Zentall (**2007**)**	RCT	36 teachers, 72 ADHD children, 72 normative children	13 teachers, 26 ADHD children, 26 normative children	Psychoeducation, Behavioral Strategies	(Face-face) 2 day sessions	Teacher behavior	Non-blinded observation, CBTC, Teacher
							Pupil behavior	

a See Appendix 3 for more detailed information on interventions and measures. KADDS = Knowledge of Attention Deficit Disorders Scale; TBQ = The Behavior Questionnaire; Conners 3-T = Conners 3-Teacher Assessment Report; YCI = Yale Children’s Inventory; BOSS = Behavioral Observation of Students in Schools; SRAQ = Self-report ADHD questionnaire; DSM-IV-TR symptom list = teacher report questionnaire based on symptom list in DSM-IV; CTRS-R = Revised Conners’ Teacher Rating Scale; PSEIA = Practice Scale of Educational Intervention Activity; K-ARS = Korean version of the ADHD Rating Scale; DSM-IV symptom list = teacher report questionnaire based on symptom list in DSM-IV; TBSS = Teacher’ Behavioral Strategies Scale; CBTC = Classroom Behavior Tally Checklist, COC = Classroom Observation Code, TRF = Teacher Report Form.

b Unpublished dissertation thesis.

c The articles by Barnett (2010) and Barnett et al. (2012) are one study with a published article and unpublished thesis reporting different detail

d The articles by Veenman et al. (2017, 2019) are one study with two published articles reporting different measures.

e clinically-diagnosed ADHD.

f fidelity measured.

A range of measures were used for the different outcomes examined in the included studies. The most proximal assessment for each study is presented in Table 4.

The mode of intervention delivery varied across studies including face-to-face training sessions and individual consultations, as well as self-directed learning from web-based materials and self-instructional booklets. Duration of training courses ranged from a single 2 hr session to a programme continuing for 18 weeks. Fidelity was only measured in five studies and training providers ranged from university trained facilitators to medical professionals, such as child and adolescent psychiatrists

In the next sections, a narrative synthesis of all included studies in the systematic review is presented first, followed by the meta-analysis from the subset of studies with sufficient data.

#### Teacher ADHD Knowledge

Teacher ADHD knowledge was measured in 17 studies (1–5, 7, 11–13, 16, 19, 21–23, 26–28; see Table 4). Of these, seven studies (4, 7, 12–13, 21–22, 26) used the full, or a modified version of the Knowledge of Attention Deficit Disorder Scale (KADDS; Sciutto et al., 2000). However, the majority of the other studies devised their own questionnaire, with only one (2) reporting validity and reliability measures. Fifteen studies (four RCTs; see Table 5) reported a statistically significant improvement in teacher ADHD knowledge in post-intervention measures, with two studies (16, 24), both RCTs, showing no significant change. Reported effect sizes were available for six studies and showed a large effect. Six of the 17 studies (7, 11–13, 19, 27; two RCTs; see appendix 4) also performed follow-up measures, ranging from 1 to 6 months post-intervention. Two studies (7, 13), both non-randomized studies, reported a significant decrease in ADHD knowledge from post-test to follow-up scores although in both cases, follow-up scores were significantly higher than pre-test scores. Two studies, comprising one RCT and one non-randomized trial (11, 19), reported no significant difference between post-test and follow-up scores, although the non-randomized trial (11) reported follow-up scores to be significantly higher than pre-test scores. One study, an RCT (12), involved a booster session two and a half weeks later at which additional measures were recorded, and reported a further significant improvement from post-test to booster scores in ADHD knowledge.

**Table 5.** Summary of Results by Outcome for Pre-Post Test Measures using Most Proximal Assessment with Effect Sizes (where reported).

Outcome measures ›	Teacher measures				Pupil measures	
Study (first author & date)	Teacher knowledge (*n* = 17)		Teacher behavior (*n* = 6)		Pupil behavior (*n* = 16)	
Aguiar (2014)	**+**	η^2^ = 0.57 (*p* < .001)				
Anto (2014)	**+**	nr				
Barbaresi (1998)	**+**	nr				
Barnett (2010, 2012)	**+**	nr	**=**	nr		
Bloomquist (1991)					**-**	nr
Both (2016)	**+**	*d* = 1.51				
Corkum (2019)					**+**	η^2^ = 0.06 (*p* = .01)
Froelich (2012)					**+**	F(1,41) = 4.98 (*p* < .031)
Gormley (2015)					*	IRD = 0.13–0.55
Kołakowski (2009)	**+**	nr				
Lasisi (2017)	**+**	*d* = 0.9				
Latouche (2019)	**+**	*d* = 2.38				
Lauth-Lebens (2016)					**+**	*d* = 1.77
Lessing (2015)					**+**	nr
Miranda (2002)	**=**	nr			**~**	nr
Mohammed (2018)					**+**	nr
Nadeau (2012)			**+**	η^2^ = 0.48 (*p* = .006)		
Obaidat (2014)	**+**	η^2^ = 0.78				
Owens (2017)			**+**	*d* = 0.33–1.12		
Park (2017)	**+**	F = 7.16 (*p* = .010)	**+**	F = 4.29 (*p* = .043)	**+**	F = 4.34 (*p* = .041)
Procaccini (2014)	**+**	nr				
Rossbach (2005)	**+**	nr			**~**	nr
Sarraf (2011)	**=**	F(1,61) = 0.14 (*p* = .71)				
Shaban (2015)					**+**	F(3, 62) = 62.98 (*p* = .001)
Shehata (2016)	**+**	nr	**+**	nr		
Syed (2010)	**+**	nr				
Tahiroğlu (2004)	**+**	nr				
Veenman (2017, 2019)					**±**	*r* = –0.074 (*p* < .01); *r* = 0.133 (*p* = .639)
Zentall (2007)			**+**	χ^2^(1, *n* = 11) = 4.28; (*p* = .039); χ2(1, *n* = 11) = 4.06, *p* = .041; χ^2^(1, *n* = 11) = 3.59, *p* = 0.049	**~**	nr

*Note.* **+** significant improvement **–** significant deterioration **=** no significant change ± outcome measures reported conflicting results.

~ incomplete data reported.

IRD = individual rate difference.

**Figure 2.**
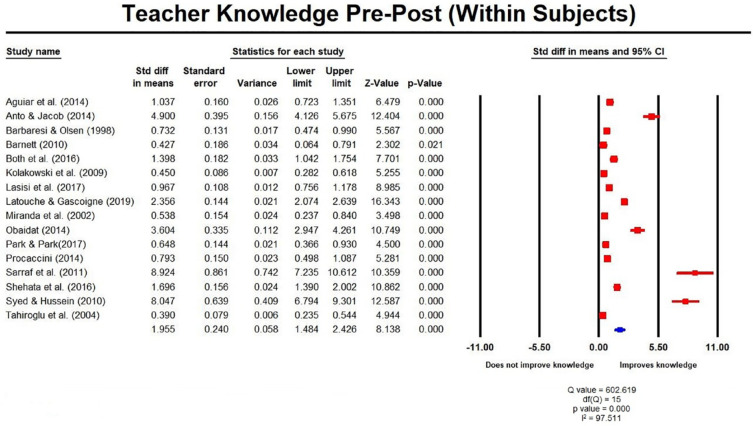
Teacher knowledge pre post measures (within subjects).

**Figure 3.**
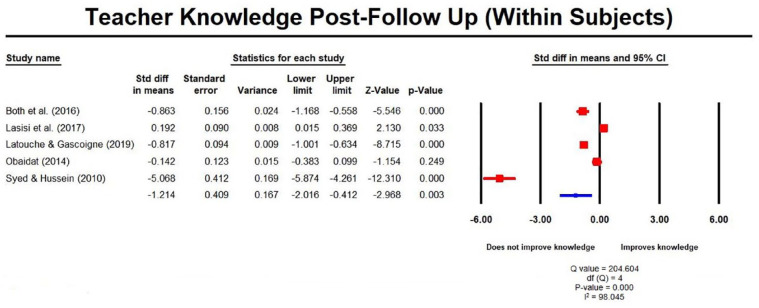
Teacher knowledge post follow up measures (within subjects).

**Figure 4.**
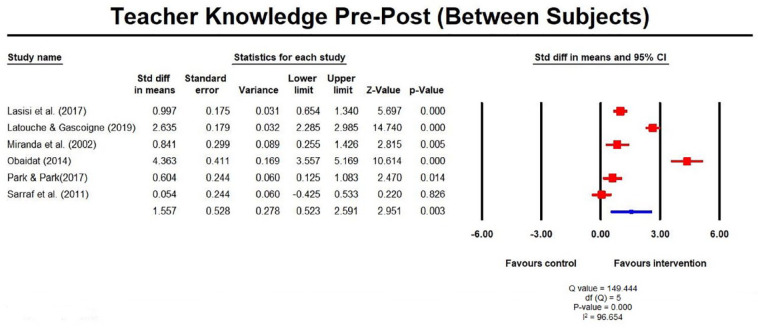
Teacher knowledge pre post measures (between subjects).

**Figure 5.**
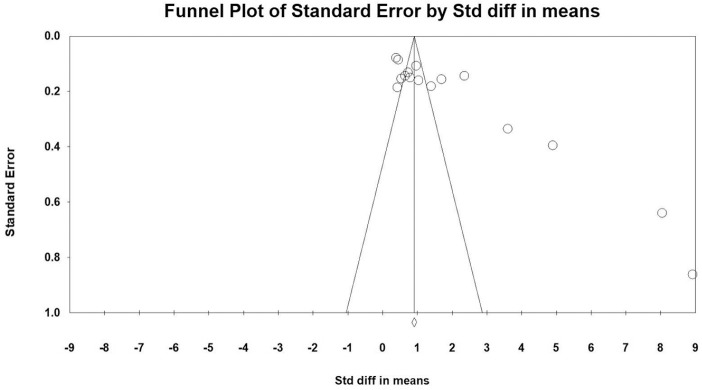
Teacher knowledge pre post measures (within subjects).

The meta-analysis of studies with within-subject designs (*n* = 16; four RCTs; Figure 2), showed that teacher training interventions produced statistically significant improvements in teacher ADHD knowledge at post-test, which were not retained at follow-up (1–6 months); SMD was 1.96 (1.48, 2.43) and ‒1.21 (–2.02, –0.41) respectively (Figure 3). For studies using between-subject designs (*n* = 6; four RCTs), the findings reflected statistically significant improvements from pre to post measures for teachers receiving the intervention compared to a control group which received no intervention; SMD was 1.56 (0.52, 2.59; Figure 4) but there was insufficient data at follow-up. Results reported for teacher knowledge did not change when only RCTs were pooled (see Supplemental Appendix 5). Publication bias was only assessed for Teacher ADHD Knowledge (Within Subjects Pre-Post Measures) as this was the only analysis that included at least ten studies (Borenstein et al., 2009, pp. 227–292; see Figure 5). The asymmetrical funnel plot and a *p* value = .0001 in the Egger’s test indicated significant publication bias (Higgins et al., 2019).

In summary, the evidence from this systematic review and meta-analysis suggests that ADHD teacher training interventions lead to a significant increase in teacher ADHD knowledge, with a large effect size. This increase in knowledge is not maintained when re-tested within 6 months of the end of the intervention although teachers do still show higher levels of knowledge than they did prior to the intervention.

#### Teacher Behavior

Six studies measured teacher behavior using self-report questionnaires (4/5, 18, 21, 26; non-randomized studies) and blinded observations (20, 31; RCTs) with only one study (4/5) showing no significant improvement at post-test. The self-report questionnaires were a mixture of study-own developed questionnaires (18, 21), and validated questionnaires by Kos (2008; The Behavior Questionnaire; reliability and validity unreported) and Azjen and Fishbein (1980; Teachers’ Behavior Strategies scale; reliability reported as acceptable (r = 0.87)). The study own questionnaires reported acceptable reliability for the scales used, although Cronbach’s alpha was only reported in the paper by Barnett (2010; α = 0.76–0.85). All studies reported post-test measures but no follow-up measures. Four studies (20–21, 26, 31) reported a significant improvement in teacher’s use of behavior management strategies, with small to large effect sizes. An additional study (27) did report a significant improvement between groups but only measured teacher behavior at post-test (no pre-test measures were taken), and only for 11 out of 49 teachers in the sample. One study (18) initially reported no significant differences post intervention, although a significant, positive change, with a large effect size, was reported following a secondary analysis introducing prior ADHD training as a covariate. Overall, teacher behavior improved post-intervention with a mixture of small to large effects but no follow-up data was available for this outcome. Additionally, the heterogeneity of teacher behavior measures meant meta-analysis of the data was not possible. Pupil ADHD-Type Behaviors. ADHD-type behaviors were measured in 13 studies using teacher rating questionnaires (8–9, 14–16, 21, 23, 25, 31; four RCTs), non-blinded observations (17, 29/30) and blinded observations (6, 10; one RCTs) as the most proximal assessment. Eight studies (8–9, 14–15, 17, 21, 25, 30; three RCTs) reported a significant positive change in ADHD-type behaviors following intervention. Effect sizes ranged from small to large. Two studies (6, 29; both RCTs) showed no significant difference at post-test. The study by Veenman et al. (2017, p. 29, 2019, p. 30) showed a significant and positive change in pupils’ ADHD-type behaviors when rated by participating teachers, but there was no significant positive change in pupil behavior when objective measures including blinded observations and actigraphy were used. Four studies (6, 8, 23, 25; three RCTs) collected follow-up measures between 2.5 weeks and 6 months. Three (8, 23, 25; two RCTs) reported a significant improvement in ADHD-type behaviors at follow-up as rated by participating teachers, with the one study reporting an effect size (23; non-randomized trial) showing a medium effect. However, the study which employed blinded observations (6; RCT), showed no significant difference at post-test or follow-up. Given the heterogeneity in interventions and study methods (e.g., follow up times), it is not possible to identify intervention characteristics that led to positive results. Additionally, the lack of blinding across studies weakens confidence in reported effects. In summary, results were mixed for pupil ADHD-type behaviors post-intervention with some studies reporting an improvement and others a deterioration.

**Figure 6.**
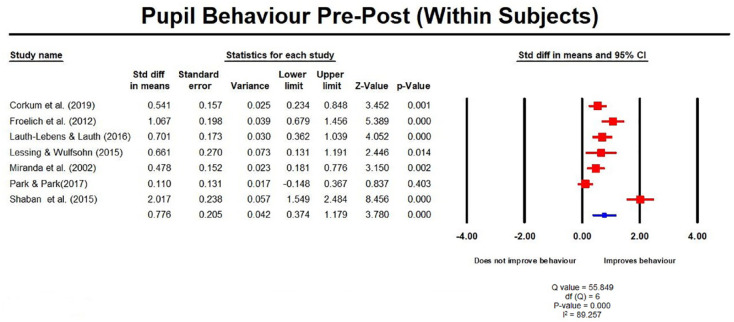
Pupil behavior pre post measures (within subjects).

**Figure 7.**
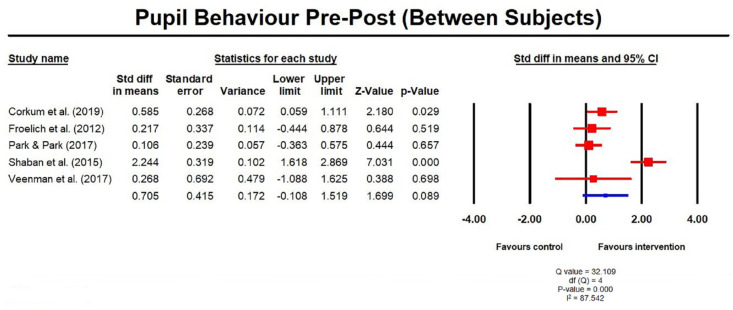
Pupil behavior pre post measures (between subjects).

The meta-analysis, which comprised three RCTs in a total of seven studies, goes some way in explaining this by identifying that, at post-test, within subject measures showed an improvement, with an SMD of 0.78 (0.37, 1.18; Figure 6) but between subject measures (three RCTs in a total of five studies) showed no significant difference, with an SMD of 0.71 (–0.11, 1.52; Figure 7). There was no difference in results when only RCTs were pooled. All of the studies in the meta-analysis (*n* = 8) used teacher ratings of pupil behavior, completed by the participating teacher in the intervention. In contrast, three studies (6, 10, 29/30) used objective measures including blinded observations and actigraphy with two of these studies (6, 29/30) reporting no improvement in pupil ADHD-type behaviors. One study (10) reported an improvement in pupil behavior but this study was a multiple baseline design with only three pupils and it was not possible to perform a meaningful comparison between this and the other studies included in this review.

**Figure 8.**
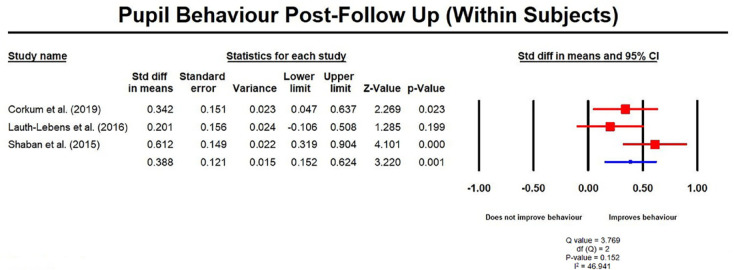
Pupil behavior post follow up measures (within subjects).

**Figure 9.**
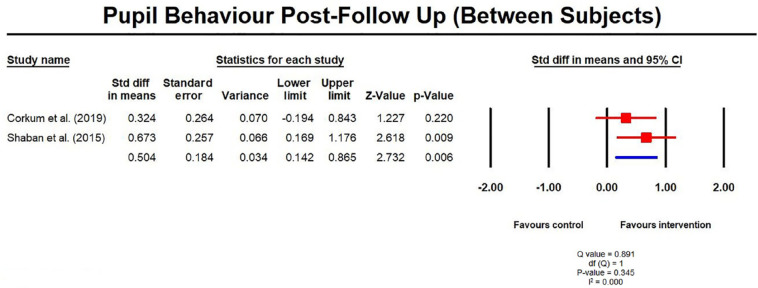
Pupil behavior post follow up measures (between subjects).

Data for effects at follow-up were only available for three studies (8, 14, 25) for meta-analysis. Interestingly, analyses revealed an overall significant improvement in pupil behavior from post-test to follow-up for within subjects (SMD = 0.39, 95% CI = 0.15, 0.62; Figure 8) and between subjects (SMD = 0.50, 95% CI = 0.14, 0.87; Figure 9), up to 6 months after the intervention had finished. This was particularly surprising for the between subject analyses, given that there had been no significant difference at post-test. On closer inspection of the data, in both cases, there was a further improvement from post-test to follow up on the two studies featuring a control group (8, 25), which had seen a significant improvement from pre-test to post-test.

In summary, the currently available evidence does not consistently suggest that ADHD teacher training interventions reduce pupils’ ADHD-type behaviors in the classrooms of participating teachers.

### Risk of Bias

**Figure 10.**
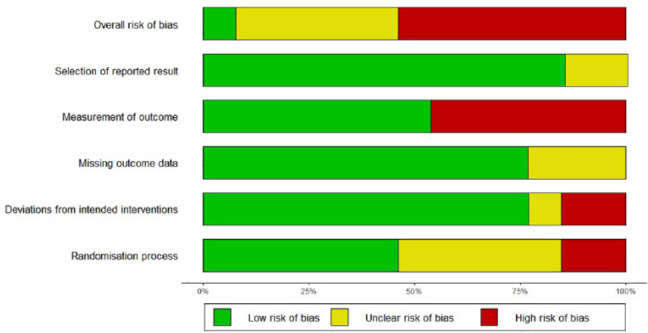
Risk of bias summary for RCTs (ROB 2.0).

**Figure 11.**
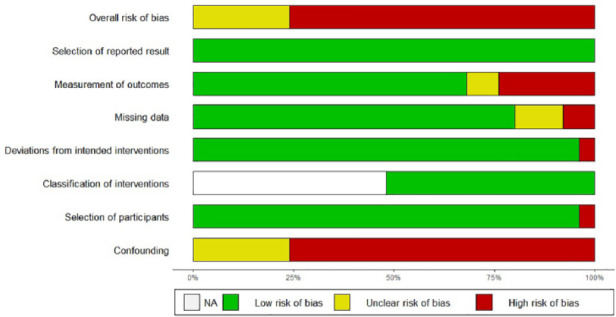
Risk of bias summary for non-randomized studies (ROBINS-I).

The intervention studies included in this systematic review and meta-analysis were predominately at risk of bias from confounding variables and the use of subjective outcome measures completed by participants, as well as a substantial lack of reporting detail on the randomization process for the randomized trials. Only four of the included studies reported using blinded outcome assessors, and none of these studies were included in the meta-analysis, highlighting the lack of reliability in the results reported. The Risk of Bias assessments (see Figures 10 and 11) highlight the medium to high risk of bias found in all studies, except one (29) which received a low risk of bias. Half of the non-randomized studies had one intervention group with no control or comparison group, and so the “Classification of interventions” domain was not applicable.

## Discussion

This study is the first to systematically synthesize the literature on the efficacy of ADHD teacher training interventions for both teacher and pupil outcomes. There is evidence that teachers play a crucial role in supporting children with ADHD in their classrooms, both in social and academic adjustment (Arcia et al., 2000; ComRes, 2017; Daley & Birchwood, 2010; Parker et al., 2013; Pfiffner & Haack, 2014), and this systematic review examined whether ADHD focused training interventions improved teachers’ knowledge of ADHD and ability to implement behavior management strategies to help pupils displaying ADHD-type behaviors.

While previous systematic reviews have explored teachers’ knowledge of ADHD (Mohr-Jensen et al., 2019) and psychoeducation for teachers (Dahl et al., 2019; Montoya et al., 2011), none have conducted a meta-analysis, nor considered effects on pupil behavior. This systematic review and meta-analysis provides a comprehensive understanding of the literature by examining the effects of specific ADHD teacher training on teachers’ ADHD knowledge, the behavioral strategies that teachers employ with pupils displaying ADHD-type behaviors, and whether there is any effect on the ADHD-type behavior of pupils in the classrooms of participating teachers. To ensure all relevant literature was included and to mitigate the risk of bias, no date or language restrictions were set, and gray literature was included in the searches.

Our study provides evidence that ADHD teacher training programs are beneficial in improving ADHD teacher knowledge immediately after training, though this should be interpreted with caution given the medium-to-high risk of bias of included studies. Importantly, this finding was consistent across almost all study designs, and intervention types. Only one study failed to detect a significant between group difference (24); this study compared two groups of teachers with both receiving information on ADHD albeit in different ways (i.e., a non-attendance ADHD psychoeducation programme was compared with an attendance-based workshop on ADHD).

Teachers in both groups showed increased knowledge of ADHD following the intervention suggesting that the mode of delivery was unimportant. One difference was noted, however. Those teachers that attended a face-to-face workshop did show a significant increase (F[1,60] = 11.3, *p* = 0.001) in knowledge of strategies to use in the classroom in comparison to those who had followed the online learning programme. The authors attributed this to more discussion of strategies in addressing particular problem behaviors (Sarraf et al., 2011).

Where reported, effect sizes were large for the increase in ADHD teacher knowledge following the intervention, but only seven out of seventeen studies reported an effect size. Our meta-analysis yielded an overall large effect size of SMD = 1.96 (95% CI = 1.48, 2.43). Therefore, it is possible that ADHD teacher training interventions increase teachers’ ADHD knowledge in a meaningful way. However, before they can be recommended, higher quality evidence is needed. Four studies (2, 19, 24, 27) reported particularly large effect sizes but each employed its own intervention and author-designed knowledge questionnaire, with a range of time frames, preventing us from identifying any possible characteristics which led to such a marked difference from the rest of the included studies. Important to note here is that the assessment of publication bias for this outcome measure suggested the likelihood of overestimation of the intervention effect (Higgins et al., 2019).

Our findings further suggest that the level of gain in ADHD knowledge following interventions was not sustained at later follow-up assessments with an overall significant decrease in knowledge (SMD = –1.21 (95% CI = –2.02, –0.41) within three months of the end of the interventions. However, knowledge still remained significantly greater than at pre-test (see Supplemental Appendix 6). It is important to note that pooling RCTs in the meta-analysis revealed no change in the direction of the effect for each analysis. Two studies did report knowledge to be sustained (12, 19) but important methodological differences need to be highlighted for these. Lasisi et al. (2017) provided a booster session of further training, two and a half weeks post intervention, in which the outcome measure was repeated. The second study (p. 19) enrolled teachers on an educational diploma, reflecting a training programme which was more time-intensive than those used in the other studies (i.e., 16 hr in total compared to the rest of the interventions being one session lasting between two and two and a half hours). Given the observed decrease in knowledge at follow-up in other studies, it is worth considering whether a more intense approach as taken by Obaidat (2014) and/or offering booster sessions is more likely to result in sustained effects at follow up, but future research is needed to address this question systematically.

Six studies reported data on teacher behaviors toward pupils with ADHD-type behaviors but the methods employed across the studies were vastly different and thus it was not possible to meta-analyze them. Our narrative synthesis of these six studies suggests that teacher training interventions can result in positive effects on teacher behavior, with only one study (4/5) showing no significant effect post-intervention. Important to note is that unlike the other studies which used either blinded observations or teacher self-report to measure change in the use of behavioral strategies, Barnett (2010) used vignettes of hypothetical scenarios. Although vignettes may be useful in allowing a direct comparison across participants’ responses to the same (hypothetical) scenario (Norcini, 2004), they also allow a sense of detachment from the situation (Poulou, 2018). Because vignettes describe hypothetical situations, these may not always relate to those experienced and of relevance to teachers in their setting. Indeed, after investigating teacher attributions for problem behavior, Lucas et al. (2009) concluded that this method using hypothetical scenarios was limited in determining how a teacher may respond to a child in real life. Although blinded observations are considered the gold standard of measuring behavior change following a workshop intervention (D’Eon et al., 2008), only two studies employed blinded observations (20, 31) with one recording very limited data (31), and neither having a control group with which to compare outcomes. The remaining studies used teachers’ self-report, thus risking biased results given teachers were not blind to intervention status and the potential expectation of change resulting from the intervention (Gualtieri & Johnson, 2005; Jerosch-Herold, 2005; Moore et al., 2019).

All studies lacked detailed information on the specific intervention components relating to behavioral strategies. However, a common factor in those studies reporting improvement in teacher behavior post-intervention was an intervention model consisting of multiple sessions over a number of weeks (6–15 weeks). This enabled teachers to use strategies in the classroom and then discuss their success or failure in subsequent meetings (Nadeau et al., 2012; Owens et al., 2017; Park & Park, 2017; Shehata et al., 2016). This enabled a problem-solving approach to address specific behaviors and adapt to an individualized model for each child (Foubister et al., 2020). One exception was the study by Zentall and Javorsky (2007) which employed a 2 day intervention. However, only post-test data for teachers’ use of positive behaviors was collected and there was no control group, rendering it difficult to make a meaningful comparison with the other studies. Given the small number of studies and the high risk of bias due to the use of teacher self-report measures, the data and evidence are currently not sufficient to suggest that teacher training interventions bring positive change in teacher behavioral management strategies. No follow-up measures were collected for this outcome and so there is currently no evidence on the long-term nature of any behavior change.

The evidence to support behavioral change in pupils with ADHD-type behaviors from this systematic review and meta-analysis is uncertain. For those studies included in the meta-analysis, teacher training interventions showed significant improvement in pupil ADHD-type behaviors compared to pre-intervention measures where SMD was 0.78 (0.37, 1.18); Figure 6) but this improvement was not seen when the intervention groups were compared to ADHD controls, where SMD was 0.71 (–0.11, 1.52); Figure 8). The direction of effect did not differ when only RCTs were pooled. It is therefore difficult to ascertain whether there would have been symptomatic improvement without intervention (Loe & Feldman, 2007). These results are reflected in the complete set of included studies for the systematic review with a range of results from a significant deterioration in pupil ADHD-type behavior (6), incomplete data from which to draw a conclusion (16, 23, 31), mixed results depending on the outcome measure used (29, 30), or a significant improvement in behavior (14–16, 21, 25) with large effect sizes where reported. Only one study used a control group of typically developing children (17), whereas the control groups in the rest of studies comprised ADHD children. This study reported a significant improvement in pupil ADHD-type behaviors for ADHD children from pre-test to post-test measures in the measurement of on-task behavior, but the intervention group did not reach the level of the normative comparison group even with these improvements (Mohammed, 2018), which has been seen in a range of ADHD behavioral interventions with participating children (Shaw et al., 2012). Furthermore, Mohammed (2018) noted that the results in his study might be due to contamination stemming from the typically developing children being in the same classrooms of participating teachers, or due to the improvement in the behavior of the ADHD pupils resulting in less distractions and a more favorable classroom environment.

Importantly, six out of the eight studies reporting an improvement in pupil ADHD-type behaviors used a teacher self-report which is reflected in the overall high risk of bias for the included studies. The two studies which provided data on blinded measures (Bloomquist et al., 1991; Veenman et al., 2019) showed a significant deterioration in pupil behavior.

Taken together, our findings suggest that while teachers who receive an ADHD training program may perceive some improvements in pupil behavior in their classrooms, the findings are limited due to non-blinded measures and lack of appropriate, controlled, comparison. Therefore, there is currently no compelling evidence that teacher training interventions lead to a reduction in pupil ADHD-type behaviors.

### Limitations

There are several limitations associated with this systematic review and meta-analysis. It was not possible to cover all existing literature as eleven requests for data were made to authors but only seven replies were received, and two sets of data were no longer available. It is possible that by selecting the outcomes to be investigated in advance, there is a risk of outcome reporting bias (Sedgwick, 2015). This risk was addressed by performing scoping searches and identifying common outcome measures used in studies investigating teacher training interventions. Differences in symptom lists, diagnostic terms and diagnostic criteria were identified and reflected in the search terms compiled by the research team. Although the risk of reporting bias was mitigated by removing all language or date restrictions from the systematic searches, by including both gray literature alongside published studies, and by including a wide range of study designs, it is possible that articles from less accessible databases were overlooked. However, the systematic searches were performed in six databases spanning medical, psychological and educational research to ensure inclusion from the breadth of literature addressing ADHD. Researcher bias through implementing the search strategy, screening of studies, risk of bias assessments and data extraction was minimized by ensuring two researchers completed each step independently, and all disagreements were resolved through discussion and consensus. There is some blurring of the lines between interventions with participating ADHD pupils, and those with participating teachers who are trained to implement behavioral strategies with pupils in their classrooms, but the inclusion criteria for this study specified that the recipients of the interventions were teachers only, and studies which reported recipients as being pupils were excluded. This may have led to some similar interventions to those included in this review being excluded according to the way in which the study was reported. It was not possible to examine differences between primary and secondary teachers due to four out of the five studies involving secondary teachers using a mixed sample of primary and secondary school teachers. This is an area that needs investigating in future research.

## Conclusion

This systematic review with meta-analysis provides some support that ADHD teacher training interventions improve teachers’ ADHD knowledge and positive behaviors toward pupils with ADHD-type behaviors, with no solid evidence to support improvements in pupil ADHD-type behaviors. The broad range of geographical locations for the included studies shows a consistency in results for different cultures and educational systems, but the high risk of bias and vast heterogeneity of interventions and measures, creates uncertainty in terms of confidence in the reported results. The strongest evidence relates to the improvement in teacher ADHD knowledge. In terms of future research, there is a strong need for high quality RCTs which investigate the specific interventions and their characteristics which produce positive outcomes for both teachers and pupils.

## Supplemental Material

sj-docx-2-jad-10.1177_1087054720972801 – Supplemental material for The Effects of ADHD Teacher Training Programs on Teachers and Pupils: A Systematic Review and Meta-Analysis Click here for additional data file. Supplemental material, sj-docx-2-jad-10.1177_1087054720972801 for The Effects of ADHD Teacher Training Programs on Teachers and Pupils: A Systematic Review and Meta-Analysis by Rebecca J. Ward, Sarah J. Bristow, Hanna Kovshoff, Samuele Cortese and Jana Kreppner in Journal of Attention Disorders

sj-docx-3-jad-10.1177_1087054720972801 – Supplemental material for The Effects of ADHD Teacher Training Programs on Teachers and Pupils: A Systematic Review and Meta-Analysis Click here for additional data file. Supplemental material, sj-docx-3-jad-10.1177_1087054720972801 for The Effects of ADHD Teacher Training Programs on Teachers and Pupils: A Systematic Review and Meta-Analysis by Rebecca J. Ward, Sarah J. Bristow, Hanna Kovshoff, Samuele Cortese and Jana Kreppner in Journal of Attention Disorders

sj-docx-4-jad-10.1177_1087054720972801 – Supplemental material for The Effects of ADHD Teacher Training Programs on Teachers and Pupils: A Systematic Review and Meta-Analysis Click here for additional data file. Supplemental material, sj-docx-4-jad-10.1177_1087054720972801 for The Effects of ADHD Teacher Training Programs on Teachers and Pupils: A Systematic Review and Meta-Analysis by Rebecca J. Ward, Sarah J. Bristow, Hanna Kovshoff, Samuele Cortese and Jana Kreppner in Journal of Attention Disorders

sj-docx-6-jad-10.1177_1087054720972801 – Supplemental material for The Effects of ADHD Teacher Training Programs on Teachers and Pupils: A Systematic Review and Meta-Analysis Click here for additional data file. Supplemental material, sj-docx-6-jad-10.1177_1087054720972801 for The Effects of ADHD Teacher Training Programs on Teachers and Pupils: A Systematic Review and Meta-Analysis by Rebecca J. Ward, Sarah J. Bristow, Hanna Kovshoff, Samuele Cortese and Jana Kreppner in Journal of Attention Disorders

sj-jpg-5-jad-10.1177_1087054720972801 – Supplemental material for The Effects of ADHD Teacher Training Programs on Teachers and Pupils: A Systematic Review and Meta-Analysis Click here for additional data file. Supplemental material, sj-jpg-5-jad-10.1177_1087054720972801 for The Effects of ADHD Teacher Training Programs on Teachers and Pupils: A Systematic Review and Meta-Analysis by Rebecca J. Ward, Sarah J. Bristow, Hanna Kovshoff, Samuele Cortese and Jana Kreppner in Journal of Attention Disorders

sj-xlsx-1-jad-10.1177_1087054720972801 – Supplemental material for The Effects of ADHD Teacher Training Programs on Teachers and Pupils: A Systematic Review and Meta-Analysis Click here for additional data file. Supplemental material, sj-xlsx-1-jad-10.1177_1087054720972801 for The Effects of ADHD Teacher Training Programs on Teachers and Pupils: A Systematic Review and Meta-Analysis by Rebecca J. Ward, Sarah J. Bristow, Hanna Kovshoff, Samuele Cortese and Jana Kreppner in Journal of Attention Disorders
