# Supplementary material for: The Effects of ADHD Teacher Training Programs on Teachers and Pupils: A Systematic Review and Meta-Analysis
Source: J Atten Disord. 2020 Dec 17;26(2):225–44. doi: 10.1177/1087054720972801 (PMC8679179; doi:10.1177/1087054720972801)
Supplement: sj-docx-2-jad-10.1177_1087054720972801 – Supplemental material for The Effects of ADHD Teacher Training Programs on Teachers and Pupils: A Systematic Review and Meta-Analysis [file sj-docx-2-jad-10.1177_1087054720972801.docx]

| **Study** | **Measure** | **Abbreviation** | **Sub-scales** | **Scales/subscales on ADHD core symptoms considered for the present meta-analysis** |
| --- | --- | --- | --- | --- |
| **Observational measures** | | | | |
| Mohammed (2018) | Behavioural Observation of Students in Schools | BOSS | On-task/ active; on-task/ passive; off-task behaviour | On-task behaviour; if not available, active on-task, passive on-task, passive off-task, disruptive off-task |
| Veenman et al. (2017) | Classroom Observation Code | COC | Interference; off-task; gross motor-all | ADHD composite score |
| Bloomquist et al. (1991) | Structured behavioural observations | - | On-task, off task/ passive, off-task/disruptive | Total; if not available, on-task behavior |
| Miranda et al. (2002) | Classroom behaviour observations | - | Off-task; disobedience; restless; disturbing teacher or peers; standing up; aggression | Total; if unavailable, off-task behaviour |
| **ADHD Rating Scales** | | | | |
| Corkum et al. (2019) | Conners 3-T teachers | Conners 3-T | Inattention; Hyperactivity-Impulsivity; Learning problems; executive function; aggression; peer relations | Inattention and/or impulsivity-hyperactivity |
| Lessing & Wolfsuhn (2015) | Conners Teacher Rating Scale Revised | CTRS-R | Hyperactivity/ Impulsivity, Perfectionism; Inattention/Cognitive Problems; Social Problems; Oppositionality; Anxious/Shy factor | ADHD index; if not available: Hyperactivity/ Impulsivity; Inattention/Cognitive Problems |
| Lauth-Lebens & Lauth (2016) | ADHD Symptoms using DSM-IV-TR analog symptom list | ADHD from DSM-IV-TR | inattention, hyperactivity, impulsivity | Total; if unavailable, inattention, hyperactivity, impulsivity |
| Froelich et al. (2012) | Yale Children’s Inventory | YCI | Hyperactivity; inattentiveness; impulsivity; oppositional defiant behaviour problems | ADHD score (combined score for hyperactivity, inattention & impulsivity) |
| Shaban et al. (2015) | Teacher Report Form | TRF | ADHD types: Inattentive; hyperactive/ impulsive; combined type | Total; if not, Inattentive; hyperactive/ impulsive; combined type |
| Park & Park (2017) | Korean ADHD Rating Scale | K-ARS | Inattention; hyperactivity-impulsivity | Total ADHD score |
