# Supplementary material for: The Effects of ADHD Teacher Training Programs on Teachers and Pupils: A Systematic Review and Meta-Analysis
Source: J Atten Disord. 2020 Dec 17;26(2):225–44. doi: 10.1177/1087054720972801 (PMC8679179; doi:10.1177/1087054720972801)
Supplement: sj-docx-3-jad-10.1177_1087054720972801 – Supplemental material for The Effects of ADHD Teacher Training Programs on Teachers and Pupils: A Systematic Review and Meta-Analysis [file sj-docx-3-jad-10.1177_1087054720972801.docx]

| **Scale name** | **Abbreviation** | **Reference** | **Measure** | **Sub-scales** | **Rater** |
| --- | --- | --- | --- | --- | --- |
| Behavioural Observation of Students in Schools | BOSS | Shapiro, E. S. (2011). *Academic skills problems workbook* (4th ed.). New York, NY: Guilford Press. | ADHD symptoms | On-task/ active; on-task/ passive; off-task behaviour | Blinded/ non-blinded observer |
| Classroom Observation Code | COC | Abikoff, H., and Gittelman, R. (1985). Classroom observation code-a modification of the Stony-Brook code. Psychopharmacol. Bull. 21, 901–909. | ADHD symptoms | Interference; off-task; gross motor-all | Blinded/ non-blinded observer |
| Conners 3^rd^ Edition Teacher Rating Scale | Conners 3-T | Conners, K. C. (2008). *Conners* (3rd ed.). Toronto, Ontario, Canada: Multi-Health Systems. | ADHD symptoms | Inattention; Hyperactivity-Impulsivity; Learning problems; executive function; aggression; peer relations | Teacher |
| Conners Teacher Rating Scale Revised | CTRS-R | Conners, C.K., Sitarenios, G., Parker, J.D. & Epstein, J.N. (1998). Revision and restandardization  of the Conners Teacher Rating Scale (CTRS-R): Factor structure, reliability, and criterion  validity. *Journal of Abnormal Child Psychology, 26*(4), 279–291. | ADHD symptoms | Hyperactivity/ Impulsivity, Perfectionism; Inattention/Cognitive Problems; Social Problems; Oppositionality; Anxious/Shy factor | Teacher |
| DSM-IV-TR symptom list | DSM-IV-TR | Lauth, G.W. & Minsel, W.R. (2014). Kölner ADHS-Test für Erwachsene (KATE). Göttingen: Hogrefe. | ADHD symptoms | Inattention, hyperactivity, impulsivity | Teacher |
| DSM-IV symptom list | DSM-IV | Rossbach, M. (2002). Entwicklung und Evaluation *eines Lehrer-Gruppentrainings zue Aufmerksamkeitsdefizit/ Hyperaktivitätsstörung (ADHS).* Unveröff. Diss., Fachbereich Psychologie, Universität Hamburg. | ADHD symptoms | Inattention, hyperactivity, impulsivity | Teacher |
| Yale Children’s Inventory | YCI | Shaywitz SE, Schnell C, Shaywitz BA, Towle VR. (1986). Yale Children's Inventory (YCI): an instrument to assess children with attentional deficits and learning disabilities. I. Scale development and psychometric properties. *Journal of Abnormal Child Psychology,14*(3), 347-364. doi:10.1007/BF00915431 | ADHD symptoms | Hyperactivity; inattentiveness; impulsivity; oppositional defiant behaviour problems | Teacher |
| Teacher Report Form | TRF | Achenbach, T. M. (1991). *Child behavior checklist/4-18.* Burlington: University of Vermont. | ADHD symptoms | ADHD types: Inattentive; hyperactive/ impulsive; combined type | Teacher |
| Korean ADHD Rating Scale | K-ARS | Kim, Y.S., So, Y.K., Noh, J.S., Choi, N.K., Kim, S.J., Koh, Y.J. (2003). Normative Data on the Korean ADHD Rating Scales(K-ARS) for Parents and Teacher.  *Journal of Korean Neuropsychiatric Association, 42*(3), 352-359. | ADHD symptoms | Inattention; hyperactivity-impulsivity | Teacher |
| Knowledge of Attention Deficit Disorders Scale | KADDS | Sciutto, M.J., Terjesen, M.D. and Frank, A.S.B. (2000), Teachers' knowledge and misperceptions of Attention‐Deficit/hyperactivity disorder. *Psychology in the Schools*, 37, 115-122. doi:10.1002/(SICI)1520-6807(200003)37:2<115::AID-PITS3>3.0.CO;2-5 | ADHD knowledge | Symptoms/ diagnosis of ADHD, treatment of ADHD, general information about the nature, causes, and outcome of ADHD | Teacher |
| Self-report ADHD Questionnaire | SRAQ  (derived from KADDS) | Sciutto, M.J., Terjesen, M.D. and Frank, A.S.B. (2000), Teachers' knowledge and misperceptions of Attention‐Deficit/hyperactivity disorder. *Psychology in the Schools*, 37, 115-122. doi:10.1002/(SICI)1520-6807(200003)37:2<115::AID-PITS3>3.0.CO;2-5 | ADHD knowledge | Symptoms/ diagnosis of ADHD, treatment of ADHD, general information about the nature, causes, and outcome of ADHD | Teacher |
| The Behavior Questionnaire | TBQ | Kos, Julie. (2008). *What do Primary School Teachers Know, Think, and Intend to Do About ADHD?* Presented at Teaching and Learning and Leadership: Australian Council for Educational Research. | Teacher behaviour | NA | Teacher |
| Practice Scale of Educational Intervention Activity | PSEIA | Kim, H.J. (2012) *Problem recognition, coping styles and educational intervention activity of teachers for the children with attention deficit, hyperactivity disorder* [master’s thesis]. Suwon: The University of Suwon. | Teacher behaviour | Adjustment of classroom environment, adjustment of teaching method | Teacher |
| The Behavioral Strategies Scale | TBSS | Ajzen, I., & Fishbein, M. (1980). *Understanding attitudes and predicting social behavior.* Englewood Cliffs, NJ: Prentice-Hall. | Teacher behaviour | NA | Teacher |
| Classroom Behavior Tally Checklist | CBTC | Zentall, S.S., & Javorsky, J. (2007). Professional Development for Teachers  of Students With ADHD and Characteristics of ADHD. *Behavioral Disorders, 32*(2), 78-93. | ADHD symptoms | NA | Teacher |
