# Supplementary material for: The Effects of ADHD Teacher Training Programs on Teachers and Pupils: A Systematic Review and Meta-Analysis
Source: J Atten Disord. 2020 Dec 17;26(2):225–44. doi: 10.1177/1087054720972801 (PMC8679179; doi:10.1177/1087054720972801)
Supplement: sj-docx-4-jad-10.1177_1087054720972801 – Supplemental material for The Effects of ADHD Teacher Training Programs on Teachers and Pupils: A Systematic Review and Meta-Analysis [file sj-docx-4-jad-10.1177_1087054720972801.docx]

*Summary of Results by Outcome for Post-Follow Up Test Measures using Most Proximal Assessment with Effect Sizes where reported*

| **Outcome measures ›** | | **Teacher measures** | | | | **Pupil measures** | |
| --- | --- | --- | --- | --- | --- | --- | --- |
|  |  | **Teacher knowledge**  **(n=17)** | | **Teacher behaviour**  **(n=6)** | | **Pupil behaviour**  **(n=16)** | |
| **Study**  **(first author & date)** | **Length of follow up** |  |  |  |  |  |  |
| Bloomquist (1991) | 6wks |  |  |  |  | **-** | nr |
| Both (2016) | 3mnths | **-** | *d*=0.77 |  |  |  |  |
| Corkum (2019) | 6wks |  |  |  |  | + | nr |
| Kolakowski (2009) | 3mnths | **=** | nr |  |  |  |  |
| Lasisi (2017) | 2.5wks  booster | **=** | nr |  |  |  |  |
| Latouche (2019) | 1mnth | **-** | nr |  |  |  |  |
| Obaidat (2017) | 1mnth | **=** | nr |  |  |  |  |
| Rossbach (2005) | 6mnth |  |  |  |  | **+** | nr |
| Shaban (2015) | 3mnth |  |  |  |  | **+** | nr |

*Summary of Results by Outcome for Pre-Follow Up Test Measures using Most Proximal Assessment with Effect Sizes where reported for those studies which report Pre-Follow Up*

| **Outcome measures ›** | | **Teacher measures** | | | | **Pupil measures** | |
| --- | --- | --- | --- | --- | --- | --- | --- |
|  |  | **Teacher knowledge**  **(n=17)** | | **Teacher behaviour**  **(n=6)** | | **Pupil behaviour**  **(n=16)** | |
| **Study**  **(first author & date)** | **Length of follow up** |  |  |  |  |  |  |
| Bloomquist (1991) | 6wks |  |  |  |  | **-** | nr |
| Both (2016) | 3mnths | **+** | *d*=0.77 |  |  |  |  |
| Kolakowski (2009) | 3mnths | **+** | nr |  |  |  |  |
| Latouche (2019) | 1mnth | **+** | nr |  |  |  |  |
| Syed (2010) | 6mnths | **+** | nr |  |  |  |  |
