# Supplementary figures and images for: The Effects of ADHD Teacher Training Programs on Teachers and Pupils: A Systematic Review and Meta-Analysis
Source: J Atten Disord. 2020 Dec 17;26(2):225–44. doi: 10.1177/1087054720972801 (PMC8679179; doi:10.1177/1087054720972801)

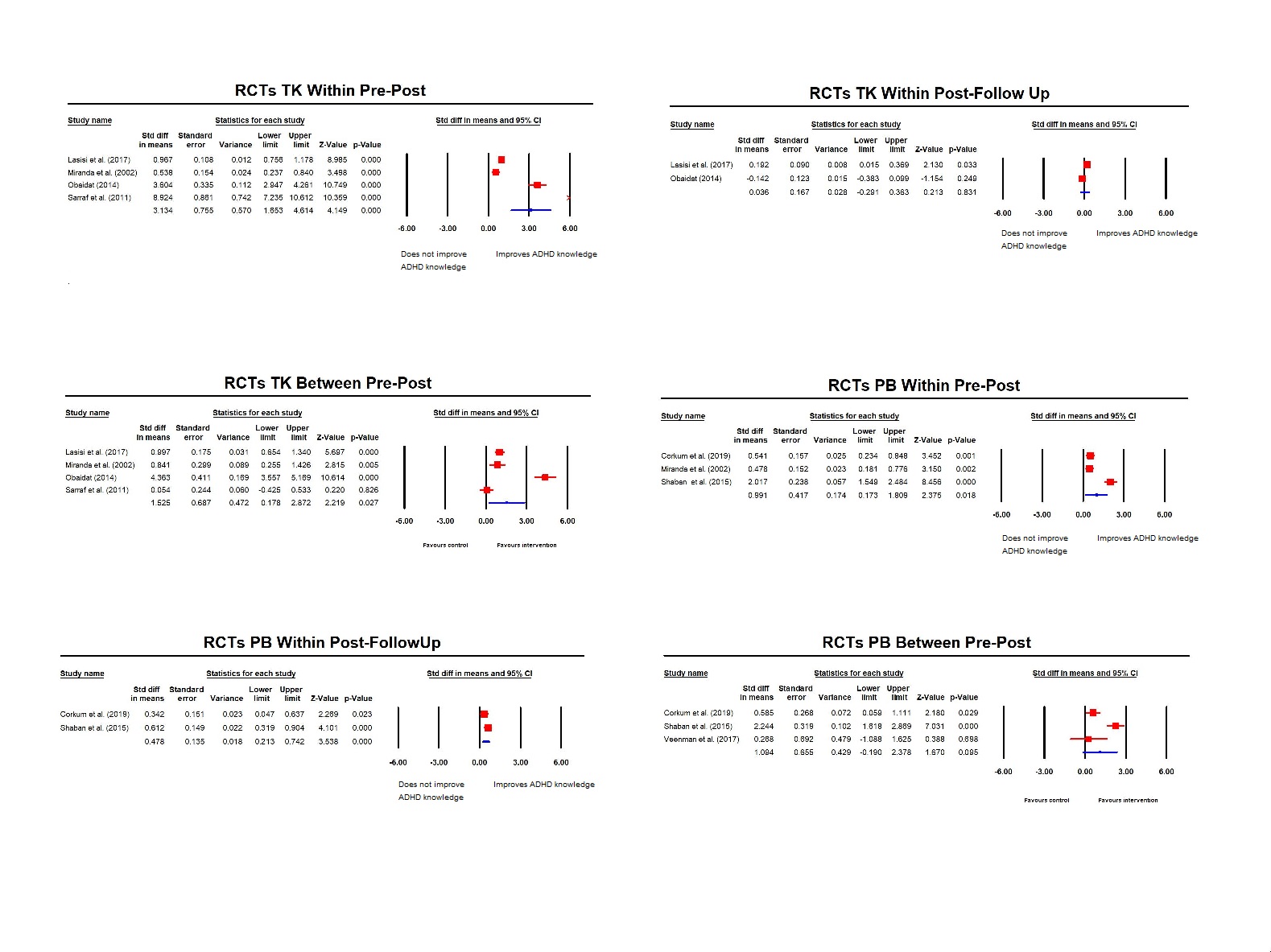

Supplement: sj-jpg-5-jad-10.1177_1087054720972801 – Supplemental material for The Effects of ADHD Teacher Training Programs on Teachers and Pupils: A Systematic Review and Meta-Analysis [file sj-jpg-5-jad-10.1177_1087054720972801.jpg]
