# Supplementary material for: The Effects of ADHD Teacher Training Programs on Teachers and Pupils: A Systematic Review and Meta-Analysis
Source: J Atten Disord. 2020 Dec 17;26(2):225–44. doi: 10.1177/1087054720972801 (PMC8679179; doi:10.1177/1087054720972801)
Supplement: Supplementary file 5 [file sj-docx-6-jad-10.1177_1087054720972801.docx]

Teacher knowledge within subjects pre-followup

Study name	Std diff in means	Standard error	Variance	Lower limit	Upper limit	Z-Value	p-Value
	Statistics for each study						
Both et al. (2016)	0.703	0.144	0.021	0.420	0.986	4.876	0.000
Lasisi et al. (2017)	1.142	0.114	0.013	0.918	1.366	10.001	0.000
Latouche & Gascoigne (2019)	1.900	0.124	0.015	1.656	2.143	15.290	0.000
Obaidat (2014)	3.876	0.357	0.128	3.176	4.576	10.848	0.000
Syed & Hussein (2010)	2.745	0.242	0.058	2.271	3.219	11.361	0.000
	2.020	0.401	0.160	1.235	2.805	5.042	0.000

Std diff in means and 95% CI

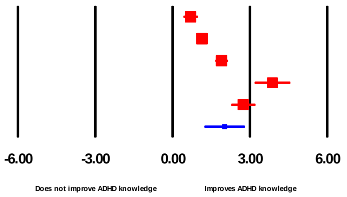

Meta Analysis
